# Bio-psycho-social characteristics and impact of musculoskeletal pain in one hundred children and adolescents consulting general practice

**DOI:** 10.1186/s12875-022-01628-8

**Published:** 2022-01-25

**Authors:** Negar Pourbordbari, Martin Bach Jensen, Jens Lykkegaard Olesen, Sinead Holden, Michael Skovdal Rathleff

**Affiliations:** 1 Department of Clinical Medicine, Center for General Practice at Aalborg University, Aalborg, Denmark; 2 Department of Health Science and Technology, Faculty of Medicine, Aalborg University, Aalborg, Denmark

**Keywords:** General practice, Musculoskeletal pain, Children, Adolescents, Characteristics

## Abstract

**Background:**

Eight percent of all child and adolescent general practice consultations are due to musculoskeletal conditions, with pain as the most frequent symptom. Despite the commonality of musculoskeletal pain, limited knowledge exists about care-seeking children and adolescents with musculoskeletal pain.

The purpose of this study was to describe characteristics of children and adolescents consulting their general practitioner with musculoskeletal pain.

**Methods:**

This is a cross-sectional study based on baseline data from the child and adolescent musculoskeletal pain cohort study (ChiBPS), carried out in 17 Danish general practice clinics. Patients aged 8–19 years who had musculoskeletal pain when consulting their general practitioner were recruited. Participants completed a questionnaire on demographics, physical activity, pain impact, psychosocial factors, and expectations of their general practitioner. Descriptive statistics were used to summarize data. Normally distributed continuous data were described using mean and standard deviation while non-normally data were described using median and interquartile range (IQR).

**Results:**

We included 100 participants (54% female, median age 13 [IQR: 12–16.5 years]). Frequent pain sites limiting activity were knee (56%), back (20%), ankle (19%), and neck (13%). Most participants (63%) consulted their general practitioner due to inability to use their body as usual, due to pain. Median pain duration at consultation was 5 months [IQR: 3 weeks-1 year]. More than a third were often/sometimes nervous (34%), worried or anxious (33%), and took pain medication (33%). Pain impeded ability to participate in sport activities at school (79%) and disturbed spare time activities (88%). Pain also made it difficult to concentrate for 58%, and to fall asleep for 38%. Only 38% expected a pain free long-term future.

**Conclusion:**

This study demonstrates the bio-psycho-social impact of musculoskeletal pain in care-seeking children and adolescents. Demographics, pain characteristics, psychosocial characteristics, and physical characteristics should be included in addressing children and adolescents with musculoskeletal pain.

**Trial registration:**

The ChiBPS study was pre-registered before participant recruitment (ClinicalTrials.gov Identifier: NCT03678922) date: 09.20.18.

**Supplementary Information:**

The online version contains supplementary material available at 10.1186/s12875-022-01628-8.

## Background

Each year, 8% of children and adolescents aged 3–17 years in the United Kingdom consult their general practitioner (GP) due to a musculoskeletal (MSK) problem [1]. Despite the ubiquity of pain, it remains poorly understood in children and adolescents and as a result may be misinterpreted as inconsequential [2]. Adolescent MSK pain has long been assumed to be innocuous with a limited impact beyond the pain experience. However, evidence indicates that adolescent MSK pain is associated with psychological distress [3], decreased quality of life [4], and a negative impact on sports participation and social activities [5, 6]. The prognosis of adolescent MSK pain is not as favorable as once assumed, and around one in every two adolescents with MSK pain continue to have pain even 1–4 years after onset [7]. This may predispose adolescents with MSK pain to chronic pain and other chronic health problems in adulthood [2].

The GP is the gatekeeper and the first point of contact in many health care systems. It is important to understand needs and impact of pain in adolescents who consult the GP for their pain. This may help support patient-centered care, which is one of the cornerstones of general practice.

Anxiety and coping among other patient characteristics may contribute to the development and maintenance of pain in children and adolescents [2]. Despite these could be relevant features to address during consultation, it is unclear how common these characteristics are among adolescents consulting general practice with pain, or the consequences on everyday lives.

We performed a systematic review investigating prognosis and prognostic factors for adolescent MSK pain [7], which informed our selection criteria and data collection. We discovered a complete knowledge gap on children and adolescents in general practice. Previous studies have primarily been in general populations, with a strong focus on pain with limited focus on psychosocial aspects of the pain experience.

The aim of this study was to explore demographics, pain features, psychosocial factors, physical activity, and expectations of children and adolescents consulting their GP with MSK pain.

## Method

### Study design and pilot work to inform the study

This cross-sectional study is based on baseline data within the child and adolescent musculoskeletal (ChiBPS) pain cohort study. The aim of the ChiBPS study is to describe prognostic factors associated to long term MSK pain among children and adolescents consulting their GP with MSK pain. The STROBE checklist for cross-sectional studies was used in reporting of the study [8].

### Setting and recruitment

#### GP clinics

From October 2018 to August 2019, one author (NP) contacted and visited general practice clinics across Denmark to introduce them to the ChiBPS study [9]. Seventeen rural and urban area clinics were included with GPs of both genders (Supplementary file 1).

#### Participants

Potentially eligible participants were invited to participate either by an employee prior to consultation, or by the consulting GP prior to or during the consultation. In each clinic, an employee or GP screened all scheduled patients for eligibility, either prior to or during consultations. The GP could choose the most suitable method in relation to the infrastructure of the clinic. Once the study was explained to the children and adolescents by the GP or employee and the decision was made to participate, they were requested to complete an electronic questionnaire (outlined in detail below). The questionnaire was hosted on a secure server at University of Aalborg (AAU) and participants were not given any specific information of the content of the questionnaire beforehand.

To be eligible, patients had to have a MSK pain complaint and this had to be mentioned by either the patient/parent or the GP as a current condition during the consultation, but not required to be the main reason for consultation. Musculoskeletal pain included pain arising from muscle, tendon, bone, and joint as per the International Association for the Study of Pain (IASP) definition [10]. The lower age limit of 8 was based on the assumption of a child’s ability to interpret/understand the questions included in our questionnaire. We did not include a pre-defined minimum or maximum pain duration as an eligibility criterion, and patients were eligible regardless of whether the current consultation was the first for their MSK complaint.

##### Inclusion criteria

- Age 8–19 years.
- Self-reported MSK pain (non-traumatic and traumatic caused by soft tissue damage, contusion or otherwise (excluding diagnosed fracture)).
- Ability to read and understand either Danish or English.

##### Exclusion criteria

- Self-reported MSK pain due to tumour, infection, or systemic and neurological causes known by either the GP or the patient/parent.

#### Data collection and management

Data was collected and managed using REDCap electronic data capture tools hosted at Aalborg University [11, 12]. REDCap (Research Electronic Data Capture) is a secure, web-based software platform designed to support data capture for research studies, providing 1) an intuitive interface for validated data capture; 2) audit trails for tracking data manipulation and export procedures; 3) automated export procedures for seamless data downloads to common statistical packages; and 4) procedures for data integration and interoperability with external sources [11, 12]. Most clinics elected to use study provided tablets to collect the data via the REDCap mobile app. If this was not possible, clinics could choose to have a link to the questionnaire sent directly to participants by a member of the research team (NP). Data was only shared with participant’s consent and the appropriate data sharing agreements in place.

All extracted data was handled in concurrence with The Danish Data Protection Agency [13] and all data extracted from REDCap and transferred to an Excel table, in an anonymised format.

#### Questionnaire and measures

The questionnaire was developed based on our systematic review, discussions with a GP reference group, and questions used in previous work [7, 14–25] (Additional file 2: Appendix 1). Our measures are divided in four sections: demographics, pain characteristics, psychosocial measures, and physical activity measures.

To ensure comprehensibility, we first piloted the questionnaire with seven 8–19-year-old children and adolescents with recent MSK pain; two girls (11 and 17 years old) and five boys (8, 9, 11, 14, and 19 years old). We received feedback regarding three statements used in the questionnaire: ‘mark the site’, ‘previous’, and ‘in what extent’, and revised these to increase comprehensibility. Otherwise, there were no major difficulties in understanding the questions and the language was considered appropriate.

#### Musculoskeletal pain

We captured MSK pain sites that participants experienced in the previous 2 weeks. Participants were able to select where they experienced pain from 33 predefined sites on a mannequin (Additional file 2: Appendix 1), and whether pain caused activity limitations or not. Activity limiting pain was defined as pain during the past 2 weeks leading to not being able to participate in play in the school yard or spare time activities [14]. Patients were able to select more than one pain locations, with more than one location of activity limiting pain being considered multi-site pain. Pain intensity was rated on a 11-point numerical rating scale from 0 to 10. Headache was not included. Our questionnaire started with three questions that was intended to ensure eligibility (see pain questions 1, 2, and 3 in Additional file 2: Appendix 1). To limit the effect of recall bias, we used a short recall period of 2 weeks on questions related to pain.

#### Data handling and statistical methods

We exported data from our questionnaires in REDCap to an Excel table and checked for any potential errors (NP). Descriptive statistics was used to summarize data (Table 1 and Table 2). Normally distributed continuous data was described using mean and standard deviation while non-normally data were described using median and interquartile range. Categorical data was described using percentages.

**Table 1:** Demographics and pain characteristics of 100, 8–19-year old care-seeking children and adolescents with MSK pain. *N* = 100. All numbers equals percentages because of the total population of 100

Demographics	
Age (median [IQR])	13 years [12–16.5]
Sex (n)	Female: 55, Male: 45
Number of siblings (median [IQR])	1 [1–2]
	Only child: 7
Position in sibling line^a^ (excluding only children and twins, *n* = 91)	First: 31
	Second: 36
	Third/fourth: 21
	Youngest: 41
**Pain characteristics**	
Pain duration (median [IQR])	5 months [3 weeks-1 year]
Pain numerical rating scale (NRS) (median [IQR])	7 [6–8]
Multi-site activity limiting pain (*n* = 53^b^)	2 sites: 23
	3 sites: 14
	4 sites: 7
	>/= 5 sites: 9
Pain episode duration (n)	< than 3 h: 34
	< than 24 h: 24
	1–7 days: 24
	> than 7 days: 18
Pain episode frequency (n)	=/> once a week: 80
	< once a week: 20

Data in Table 1 are based on 97–100% replies.

^a^fifth child, *n* = 3, twins, *n* = 2. ^b^five participants reported only one pain site and this was non-activity limiting – as answer to pain question 3, of these one of the sites were the jaw. (ID 40, 42, 51, 57, 90)

**Table 2:** Psychosocial and physical characteristics of 100, 8–19-year old care-seeking children and adolescents with musculoskeletal pain. *N* = 100. All numbers equals percentages because of the total population of 100

Psychosocial characteristics	
Pain outside school hours (n)	97
Nervous (n)	Often/sometimes: 34
	Seldom/never: 66
Worried or anxious (n)	Yes: 33, No: 32, I don’t know: 35
Low self-esteem (n)	Yes: 7, No: 78, I don’t know: 15
Believe in God (n)	Yes: 36, No: 35, I don’t know: 29
Difficult to fall asleep because of pain (n)	38
Tired during the day (n)	57
Have a job (n)	33
Know the cause of pain (n)	58
Expect the GP to prescribe pain medication (n)	8
Pain affects my concentration (n)	58
Take pain medication for pain (n)	33
Frequency of pain medication (n)	Once/month: 13
	Once/week: 12
	More than once/week: 6
	Every day 1
Know the name of pain medication, *n* = 26	Paracetamol: 17
	NSAID^a^: 1
	Paracetamol and NSAID: 8
**Physical activity characteristics**	
Physical active besides school hours times/week, *n* = 80^b^	0: 0
	1: 11
	2–3: 39
	4–6: 16
	> 6: 5
Screen time/other activities mostly sitting down outside school hours hours/day^c^ (n)	0: 2
	1–2: 36
	3–6: 49
	>/= 7: 7
Pain disturbs (separate questions) (n):	a walk longer than 1 km: 70
	my spare time activities: 88
Pain makes it difficult to (more than one option could be ticked) (n):	stand in a queue for 10 min.: 36
	carry my school bag to school 22
sit on a chair for a 45-min. Lesson 31	bend down to put on my socks: 33
do sport activities at school 79	run fast to catch a bus: 67

Data in Table 2 are based on 97–100% replies; question concerning screen time had the lowest reply percentage.

^a^NSAID: non-steroidal anti-inflammatory drug. ^b^incl. One answer to; sometimes once other times 3, 1–2 times, 1–3 times, and 4–7 times, two answers 3–4 times, three answers: 3–5 times. ^c^Excl. one answer: 1-3 times, many times, and all the time and three answers: 2–3 times

## Results

### Study group characteristics

A total of 124 children and adolescents were recruited from 17 GP clinics. Of these, 24 were excluded; six due to missing consent, fifteen due to incomplete/cloned questionnaire, and three due to lack of fulfilment of eligibility criteria resulting in 100 participants (Table 1 and Table 2). The primary activity limiting pain sites were knee (56%), ankle (18%), back (14%), heel (12%), foot (12%), and neck (9%) with a median pain intensity of 7 (IQR 6–8). The median pain duration was 5 months [3 weeks-1 year]. Multi-site activity limiting pain was reported by 53%. Almost all children and adolescents had pain outside school hours (97%) and were disturbed by their pain during their hobbies (88%).

**Fig. 1:**
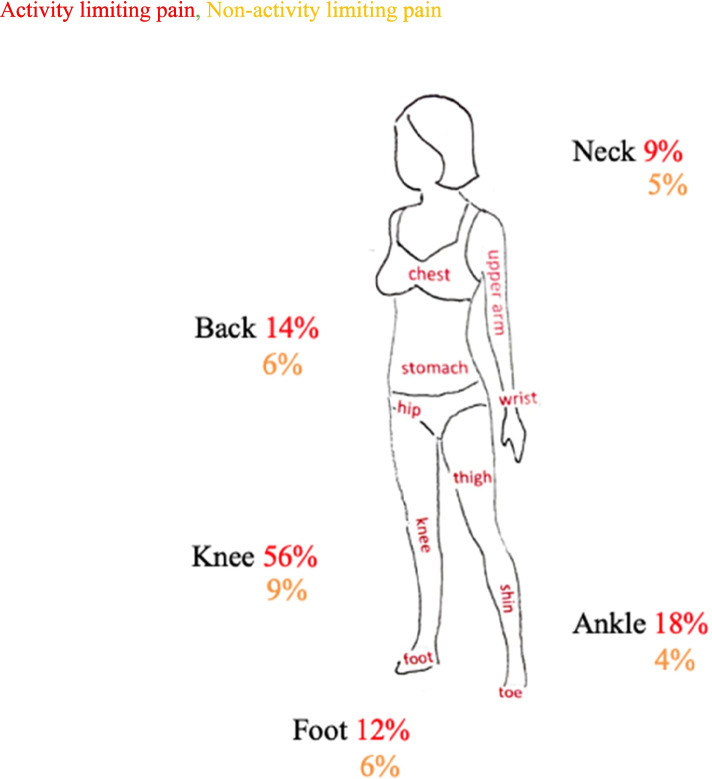
Differentiating between activity limiting and non-activity limiting pain sites. The data depicts participants with the most frequent pain sites, stratified as activity and non-activity limiting pain. Data based on all participants, *n* = 100. Activity limiting and non-activity limiting pain are not mutual exclusive. One participant experienced activity limiting right sided knee pain and non-activity limiting left sided knee pain

**Fig. 2:**
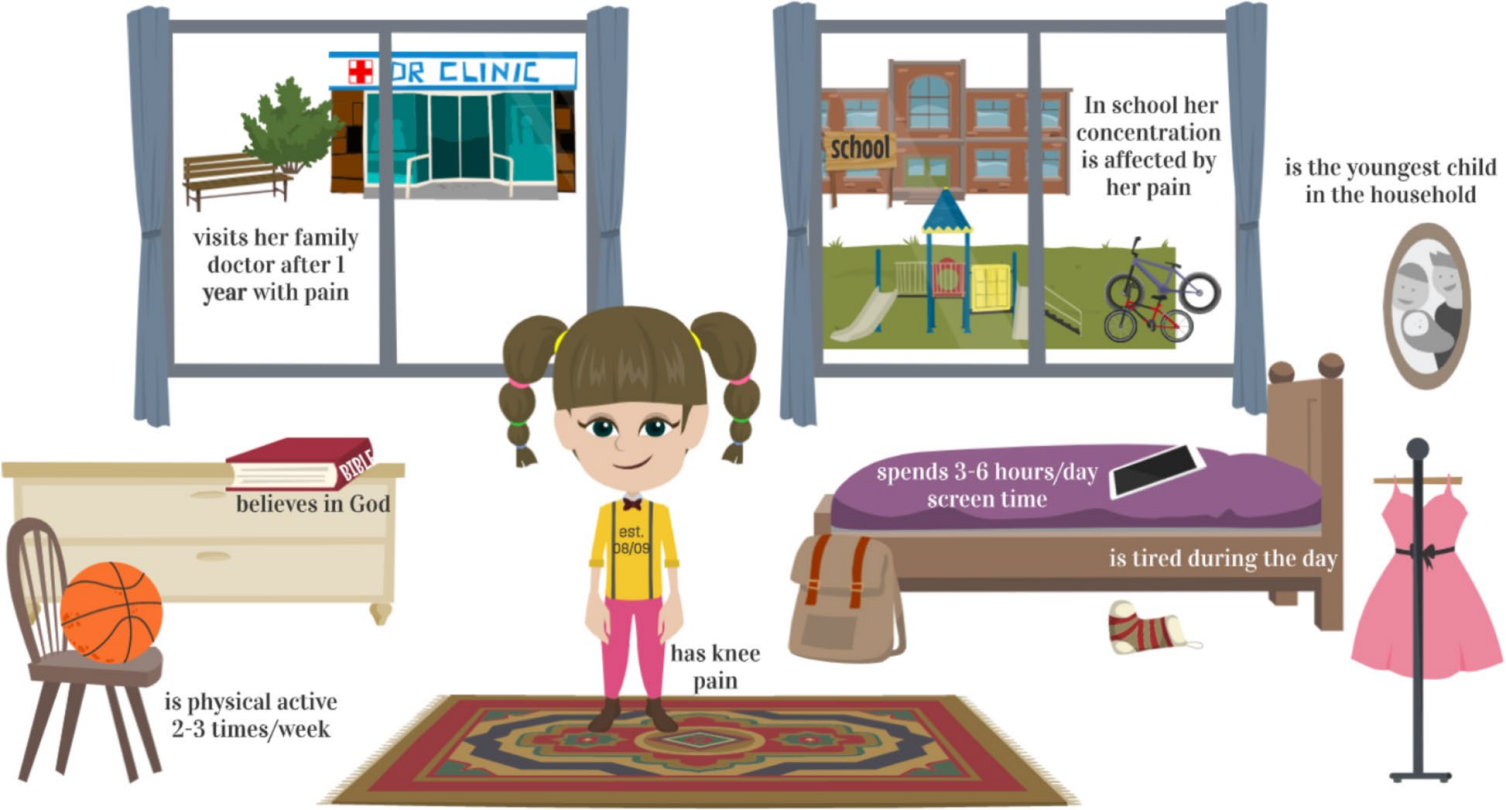
Common characteristics of a GP care-seeking 8–19-year old with musculoskeletal pain. A short story about a young girl with pain. A typical Danish child or adolescent with musculoskeletal pain is a 12 or 13 year-old girl. She has pain in her knee and in at least one more body part. She visits her general practitioner because she cannot use her body as usual due to pain and she decides to do so after having had pain for one year with pain episodes occurring as frequently as once every week. In her household she is the youngest of two children. In school her concentration is affected by her pain, and she goes on with her day feeling tired, but after school she is active in sports 2–3 times a week, even though her pain disturbs her spare time activities. During a typical day, she spends 3–6 h looking at a screen. She believes in God. When her day is over, and it is time for her to turn in she goes to bed knowing what causes her pain. Data is based on all participants, *n* = 100 including both activity limiting and non-activity limiting pain. Cut off limit is defined at a minimum of 31% of all participants for inclusion of the characteristics included in this figure

Figure 1 highlights the difference in activity limiting and non-activity limiting pain by pain sites, with knee pain the most frequent site of both. Figure 2 visualizes the common characteristics of a typical Danish child or adolescent with MSK pain, including demographics, physical activity, family pattern and pain impact on school.

## Discussion

### Main findings

Knee and ankle pain were the two most common activity-limiting pain sites among a care-seeking population of adolescents with MSK pain in general practice. Fifty-three percent experienced multi-site pain. Overall, 33% had used pain medication during the past 2 weeks and 13% used it at least once a month. Median pain duration was 5 months and a range of different functional and social limitations due to pain were reported.

### Findings in relation to existing literature

Previous research from UK revealed that 8% of an adolescent population seek care from their GP due to MSK conditions each year [1]. It has so far been unknown how large the impact of pain is among this primary care population. Previous research is mainly in secondary care populations or in school-based populations. Studies generally observed a longer pain duration than the current study (often > 12 months) [7], with a high proportion who had previously contacted a health care practitioner [26]. The proportion experiencing multi-site pain in our study was lower compared with previous studies [27]. Multi-site pain seems to develop over time with increasing pain duration [28]. This could indicate that our population contacts general practice early in the pain development. Early intervention has been proposed to improve long-term outcomes due to duration of pain complaints, multi-site pain and psychological symptoms associated with a poor prognosis [7]. Most of our sample suffered from either back or knee pain which aligns with the findings from UK general practice [1] and school-based populations in Denmark [29].

### The impact of pain

Konijnenberg et al. [30] found approximately 50% school absence because of pain. We found 22% reported difficulties in carrying their school bag to school, 31% had difficulties sitting for a 45-min lesson, and 58% reported a negative effect of pain on their concentration. The most common causes for consultation in this study were limitation in the habitual use of the body (64%), wanting the pain to stop (59%), and worrying about the cause of pain (55%). This is similar to research showing that pain intensity and activity limiting MSK pain were important drivers for seeking care among adolescents with pain complaints [31, 32].

### Explanation of findings

Our findings underline the need to consider psychological and social factors since female sex (55%), pain duration more than 1 year (24%), feeling anxious (33%), daytime tiredness (57%), more than 6 non-school hours of sitting down/day (7%) and smoking (2%) are associated with an increased risk of a poor prognosis [7]. Co-occurring pain, psychological and social factors in general practice should be considered treatment-targets and we recommend questioning any recent events in the family or surroundings, that could potentially have an impact on the child since there is a lack of knowledge on the effect of these modifiable risk factors.

### Care-seeking behaviour

Despite back pain affect 33% of children in school-based populations, only 6 % of them seek care for their back pain [33]. Care-seeking behaviour in children is hence uncommon. This could indicate that years of pain duration push for a consultation rather than a wait and see approach.

Previous research suggest that 50–65% of children and adolescents have MSK pain 1–4 years after onset [7] whereas 14% of our population reported a pain duration of 1 year prior to the current consultation. Both numbers call for clinical implications, since long-term MSK pain condition can push toward a more progressive investigation by the GP. General practitioners commonly prescribe a wait and see treatment for MSK pain [34, 35].

### Implications for practice and future research

Our results underline the bio-psycho-social impact of MSK pain in care-seeking children and adolescents. Importantly, the results reveal the wide-reaching impact on carrying a school bag, the concentration, and the negative impact on leisure time activities. MSK pain in adolescents was once considered a benign self-limiting condition with limited impact beside the actual pain experience. These results underline that GPs need to be cognizant of the widespread impact and challenges these young individual’s experience.

### Strengths and limitations of the study

Our study data was drawn from a nationwide cohort, representative of the Danish population in age, sex, and environment (Table 1, Table 2, Supplementary file 1). It is unclear if our findings are generalizable to other countries with different health care sectors, differences in care-seeking behaviour and cultural differences. We used validated questions when possible, and piloted the survey to ensure that children and adolescents understood the questions. Due to the commonality of pain, we collected data on pain that affected their typical activities and otherwise pain. This distinction is important and ensure we can differentiate pain with and without an impact on the individual. Self-report measures of pain and other factors may be affected by recall bias. To limit recall bias we used a short recall period of 2 weeks. Due to the small number of adolescents included, we did not stratify pain characteristics or pain impact into specific body sites. In this study we used “alcohol consumption” more than once per month, while previous studies have used the term “occasional use” [7]. This may make a direct comparison difficult. We did not collect data on NSAID intake for specifically MSK pain and are not able to exclude dysmenorrhea as a common pain condition among menstruating female adolescents.

## Conclusion

Two thirds of children and adolescents consult their GP because of limitations in the habitual use of their body due to pain. One third of children and adolescents are nervous or worried/anxious and more than half report their concentration is affected by their pain. These findings and other bio-psycho-social factors are important in addressing children and adolescents with musculoskeletal pain as they represent co-occurring conditions.

## Supplementary Information

**Additional file 1.**

**Additional file 2.**

## Data Availability

The datasets used in the current study are available from the corresponding author on reasonable request.

## Abbreviations

GP: General practitioner
MSK: Musculoskeletal
