# Supplementary material for: Bio-psycho-social characteristics and impact of musculoskeletal pain in one hundred children and adolescents consulting general practice
Source: BMC Prim Care. 2022 Jan 25;23:20. doi: 10.1186/s12875-022-01628-8 (PMC8790922; doi:10.1186/s12875-022-01628-8)
Supplement: Supplementary file 1 — Additional file 1. [file 12875_2022_1628_MOESM1_ESM.docx]

**Supplementary file 1**

Recruited GP clinics who recruited participants for this study and their locations in the country of Denmark,. Inhabitants in the community where the clinic is located, recruitment done by one single GP (S) or multiple (M), and gender of these indicated as female (f), male (m) or both (f/m).

Malling 6.194 S, f

Silkeborg city 46.923 M, f/m

Århus city 65.969 M, f/m

Hobro city 12.130 S, f

Svenstrup 4.566 M, f/m

Mariager city 2.537 M, f/m

Nibe city 5.217 M, f/m

Vodskov 4.566 S, m

Nørresundby 23.546 M, f

Sæby 8.871 M, f/m

Frederikshavn 23.124 M, f/m f/m

Bjergby 860 S, f

Odense city 180.302 S, m

Nors 1.051 S, m

Thisted city 13.536 M

Vrå 2.474 M, f/m

Hjørring 25.780 M, f/m
