# Supplementary material for: Bio-psycho-social characteristics and impact of musculoskeletal pain in one hundred children and adolescents consulting general practice
Source: BMC Prim Care. 2022 Jan 25;23:20. doi: 10.1186/s12875-022-01628-8 (PMC8790922; doi:10.1186/s12875-022-01628-8)
Supplement: Supplementary file 2 — Additional file 2. [file 12875_2022_1628_MOESM2_ESM.pdf]

Questionnaire for children and adolescents with musculoskeletal pain

Hi there,

thanks a lot for your participating in my research project:

Children and adolescents with musculoskeletal pain: prognosis, ethnicity, and long term pain (MPU 20-2017).

I appreciate it.

If you should have any questions, feel free to contact me on 27914224.

Best regards, Negar, medical doctor

First, I would like to know whether you have turned 17 years old?	◯ Yes ◯ No
I consent voluntarily to my child's participation in this research project.	__________________________________
I have received written and oral information of the project.	◯ Yes ◯ No
Name of the person custody lies with: (One name is sufficient).	____________________________
Do you wish to be informed of the results of the project and possible consequences for your child?	◯ Yes ◯ No
I consent voluntarily to participate as a participant in this research project:	__________________________________
Do you wish to be informed of the results of the project and possible consequences for you?	◯ Yes ◯ No
Back to you (the patient). What is your name?	____________________________
What is your name?	____________________________
What is your CPR. no.?	____________________________

Mobile phone no.
Please write your parent's phone no. if you don't have one yourself ______________________

What sex are you? ◯ Girl ◯ Boy

What is the name of your general practitioner or his/her clinic? ______________________

The next questions concern your pain.

Below is a drawing of your body with names of the different parts of the body, where you can experience pain or feel hurt.

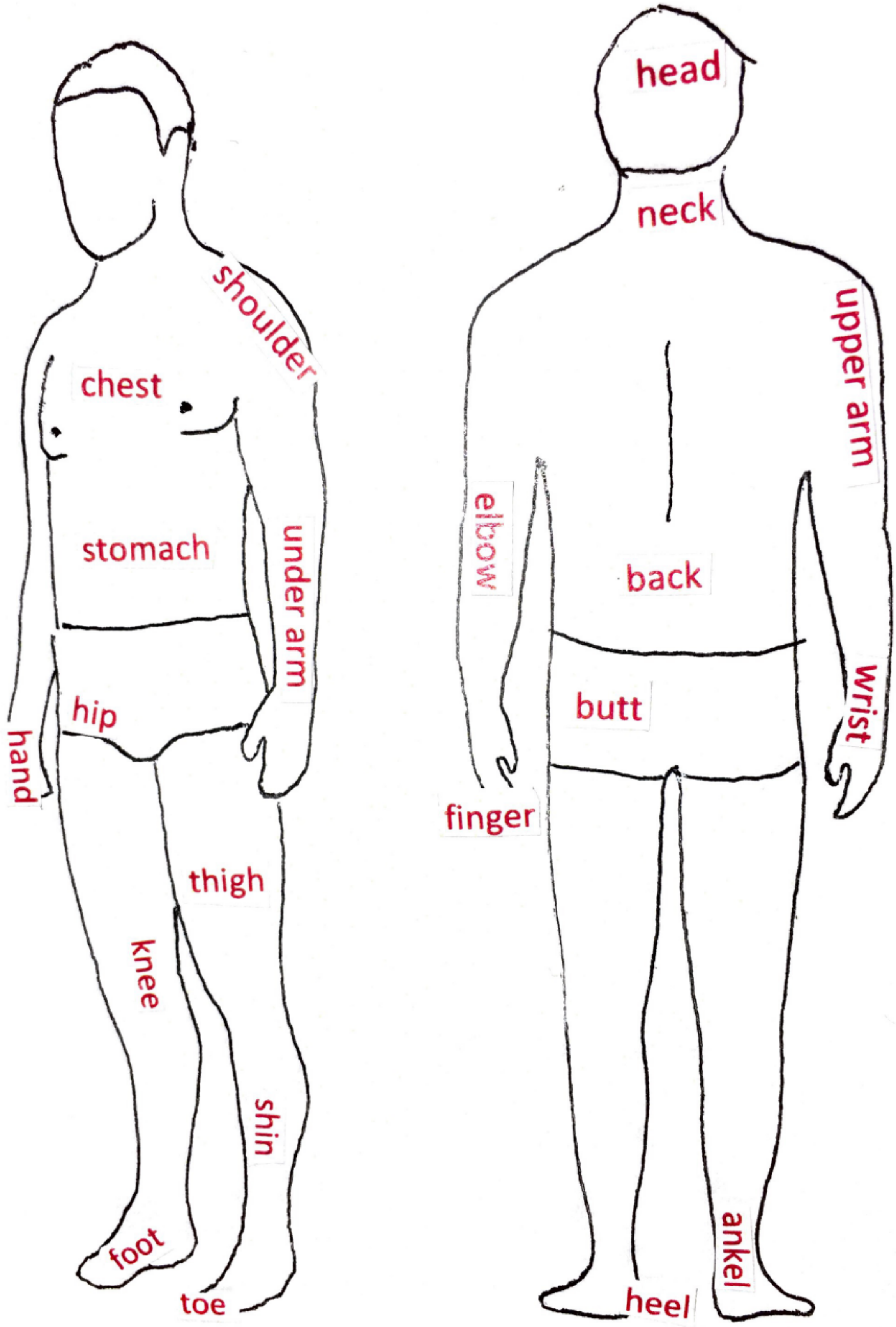

Below is a drawing of your body with names of the different parts of the body, where you can experience pain or feel hurt.

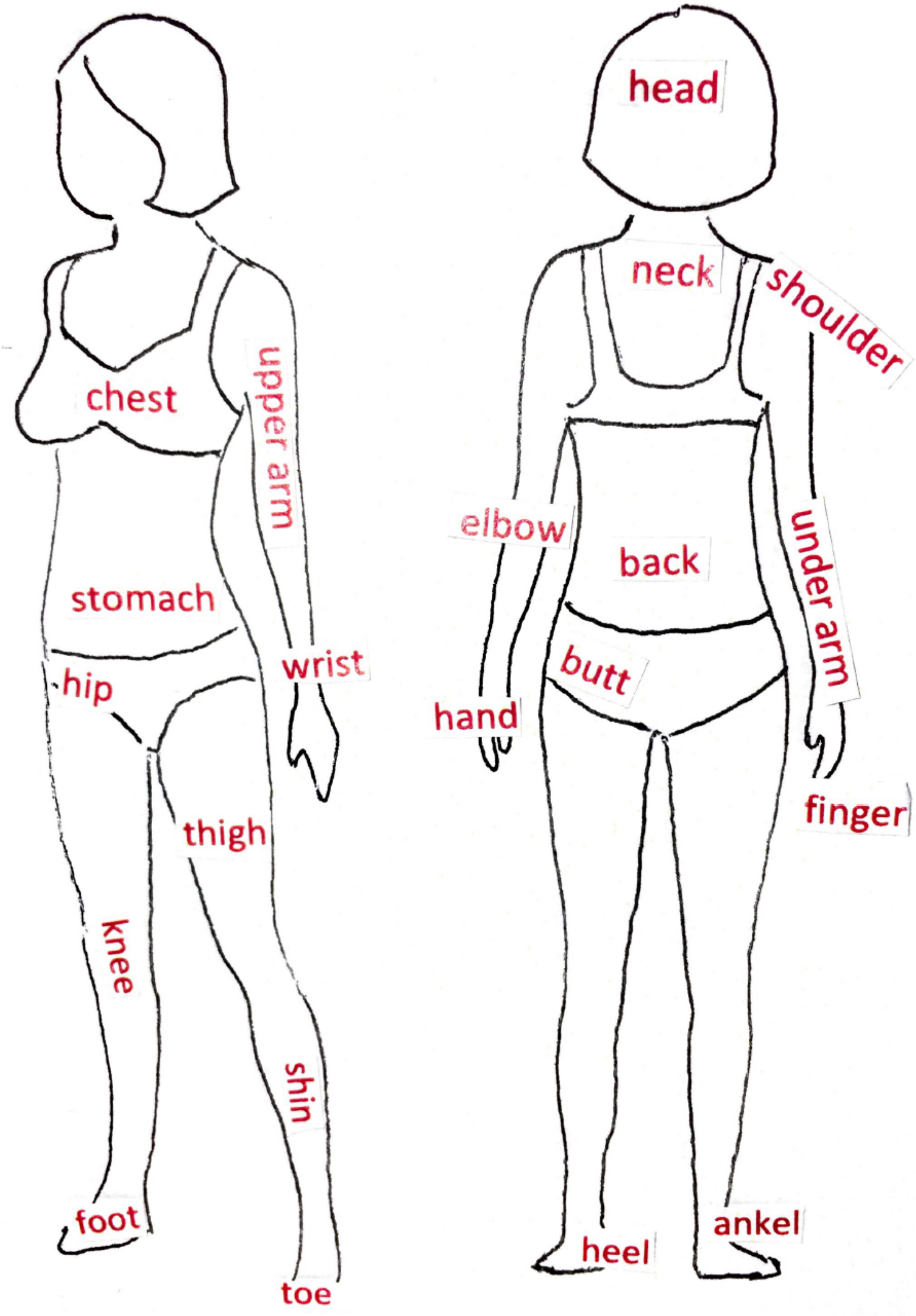

Have you experienced pain in the past two weeks that have lead you to not being able to participate in play in the school yard or sparetime activities (ex. football or other spare time activity)?	◯ Yes ◯ No
Please mark the areas of your body where you have pain during the past two weeks.<br>If you have pain in more than one area of the body, it is important that you mark all the places with pain.<br><br>Example: If you have pain in both the right knee and left foot, you must mark "right knee" and "left foot".	☐ Head<br>☐ Neck<br>☐ Right shoulder<br>☐ Left shoulder<br>☐ Right side of the chest<br>☐ Left side of the chest<br>☐ Right upper arm<br>☐ Left upper arm<br>☐ Right elbow<br>☐ Left elbow<br>☐ Right under arm<br>☐ Left under arm<br>☐ Right wrist<br>☐ Left wrist<br>☐ One or more fingers on the right hand<br>☐ One or more fingers on the left hand<br>☐ Right hip<br>☐ Left hip<br>☐ Back<br>☐ Right hip<br>☐ Left hip<br>☐ Right knee<br>☐ Left knee<br>☐ Right shin<br>☐ Left shin<br>☐ Right ankel<br>☐ Left ankel<br>☐ Right heel<br>☐ Left heel<br>☐ Right foot<br>☐ Left foot<br>☐ One or more toes on the right foot<br>☐ One or more toes on the left foot
The next question is whether you have pain in other areas of your body, different from the ones selected in previous question.<br><br>Do you have pain in other areas of the body?	◯ Yes ◯ No

Where?	☐ Head<br>☐ Neck<br>☐ Right shoulder<br>☐ Left shoulder<br>☐ Right side of the chest<br>☐ Left side of the chest<br>☐ Right upper arm<br>☐ Left upper arm<br>☐ Right elbow<br>☐ Left elbow<br>☐ Right under arm<br>☐ Left under arm<br>☐ Right wrist<br>☐ Left wrist<br>☐ One or more fingers on the right hand<br>☐ One or more fingers on the left hand<br>☐ Right hip<br>☐ Left hip<br>☐ Back<br>☐ Right hip<br>☐ Left hip<br>☐ Right knee<br>☐ Left knee<br>☐ Right shin<br>☐ Left shin<br>☐ Right ankel<br>☐ Left ankel<br>☐ Right heel<br>☐ Left heel<br>☐ Right foot<br>☐ Left foot<br>☐ One or more toes on the right foot<br>☐ One or more toes on the left foot
If you have pain in an area of the body, not named on the drawing above, please write it here:	______________________________
Mark the statements to the right, that are true about your pain. You may mark more than one.	☐ It can easily be ignored<br>☐ It affects my concentration<br>☐ Sometimes I have to take pain medication<br>☐ Sometimes I can't attend school because of the pain<br>☐ None of the above
On the scale of 0 to 10 to the right, where 0 is no pain and 10 is the worst possible pain, mark the number that best represents your pain.	◯ 0<br>◯ 1<br>◯ 2<br>◯ 3<br>◯ 4<br>◯ 5<br>◯ 6<br>◯ 7<br>◯ 8<br>◯ 9<br>◯ 10
How long have you had your current pain?	______________________________
Are you familiar with the cause for your current pain?	◯ Yes ◯ No

What is the cause for your current pain (ex. a fall, a hit or other)?	__________________________
How long does a pain episode usually last?	◯ Less than 3 hours<br>◯ Less than 24 hours<br>◯ 1-7 days<br>◯ Longer than 7 days
How often do have a pain episode?	◯ At least once a week<br>◯ Less than once a week
Do you expect to be free of your current pain in the future?	◯ Yes, in the near future<br>◯ Yes, in the long-term future<br>◯ No
Do you take painkillers?	◯ No<br>◯ Yes, I take painkillers and know the name of them<br>◯ Yes, I take painkillers, but do not know the name of them
Please write the name of the painkillers that you take	__________________________
How often do you take painkillers?	◯ Once a month<br>◯ Once a week<br>◯ More than once a week<br>◯ Every day
Is your pain radiating to your legs or arms?	◯ Yes ◯ No

The next questions concern your doctors appointment today.

Why did you come to your doctor today?<br>You may choose more than one answer.	☐ I want my pain to stop<br>☐ I am worried about the cause of my pain<br>☐ My family made me come<br>☐ I have a personal problem<br>☐ Because of my pain, I can't use my body as I used to<br>☐ None of the above
Do you expect your doctor to give you medication for your pain?	◯ Yes, I would like that<br>◯ No
Is this your first visit to your general practitioner concerning your current musculoskeletal pain condition?	◯ Yes ◯ No
This is visit number:	__________________________
Do you feel worried or anxious?	◯ Yes<br>◯ No<br>◯ I don't know
Do you believe, that you have low self-esteem?	◯ Yes<br>◯ No<br>◯ I don't know

Do you believe in God?	◯ Yes<br>◯ No<br>◯ I don't know
Is it difficult to fall asleep because of pain?	◯ Yes ◯ No
Is it difficult to sit during a lesson?	◯ Yes ◯ No
Does your pain disturb a walk longer than 1 km?	◯ Yes ◯ No
Does your pain disturb your physical exercise?	◯ Yes ◯ No
Does your pain disturb your hobbies?	◯ Yes ◯ No
Do you have pain outside school hours?	◯ Yes ◯ No
Are you tired during the day?	◯ Yes ◯ No
Does your pain make any of the following activities difficult? You may mark more than one.	◯ Reaching up to get a book from a high shelf<br>◯ Carrying your school bag to school<br>◯ Sitting on a school chair for a 45-min. lesson<br>◯ Standing in a queue for 10 min<br>◯ Sitting up in bed from a lying position<br>◯ Bending down to put your socks on<br>◯ Standing up from an armchair at home<br>◯ Running fast to catch a bus<br>◯ Sport activities at school<br>◯ None of the above

The next questions concern both during school time and when you are not at school.

Outside school hours, how many hours a day do you spend watching TV/tablet/mobile phone/computer or do other activities when mostly sitting down?<br>If you spend less than 1 hour a day, please write 0.	__________________________
How much do you sleep on average per night?	◯ 7 hours or less<br>◯ 8-10h<br>◯ More than 10 hours
Are you physical active besides school hours (ex. dance, fitness, swimming, or similar)?	◯ Yes ◯ No
How many times a week do you do sport?	__________________________
Do you drink alcohol?	◯ Yes ◯ No
How often do you drink alcohol?	◯ More than once a month<br>◯ Approximately once a month<br>◯ Less than once a month<br>◯ I have only had alcohol one time<br>◯ I have never had alcohol
Do you smoke cigarettes?	◯ Yes ◯ No

How many cigarettes do you smoke a day?	◯ I don't smoke cigarettes every day<br>◯ 1-4 cigarettes a day<br>◯ 5-9 cigarettes a day<br>◯ More than 9 cigarettes a day
Do you have a job?	◯ Yes ◯ No
How would you generally describe your physical activity in your job?	◯ Mostly sedentary work that does not require physical exertion<br>◯ Mostly standing or walking work, which otherwise does not require physical exertion<br>◯ Standing or walking work with some lifting or carrying work<br>◯ Heavy or fast work which is physically strenuous<br>◯ I don't know

The following questions are general questions about you.

How often do you feel nervous?	◯ Often or sometimes<br>◯ Seldom or never
Which zip code(s) do you live in?<br>If you live in several places please write both zip codes.	____________________________<br>(If you live more than one place, please write both zip codes)
In which country were you born?	◯ Danmark ◯ Other country
In which other country were you born?	____________________________
How many years have you lived in Denmark?	____________________________
How many siblings do you have (including non-biological or siblings with a different mother or father than yours)?	◯ I don't have any siblings / I am an only child<br>◯ 1<br>◯ 2<br>◯ 3<br>◯ 4<br>◯ 5<br>◯ 6<br>◯ More than 6
Which number are you in your group of siblings?	____________________________
What do you feel the most as?	◯ Danish<br>◯ Danish with foreign background<br>◯ Foreigner<br>◯ I don't know<br>(Please answer the question, whatever your country of birth.)
How large a part of your friends have immigrant background or were not born in Denmark?	◯ None<br>◯ Almost none<br>◯ Almost all<br>◯ All

The last questions concern the development of your body.

Below is an image of the girl's body, and how it can look from the age 8 to 19 years.

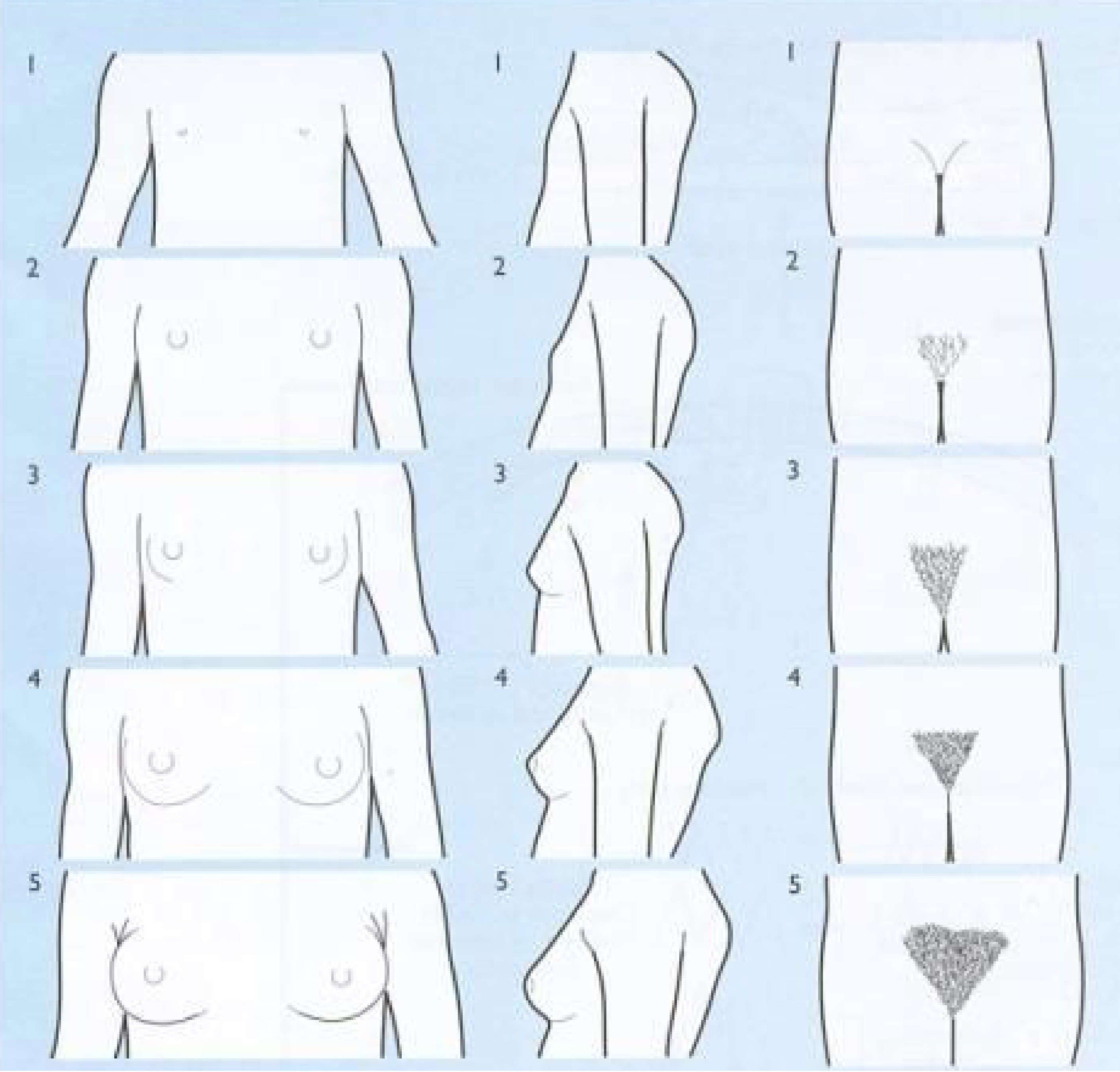

Below is an image of the boy's body, and how it can look from the age 8 to 19 years.

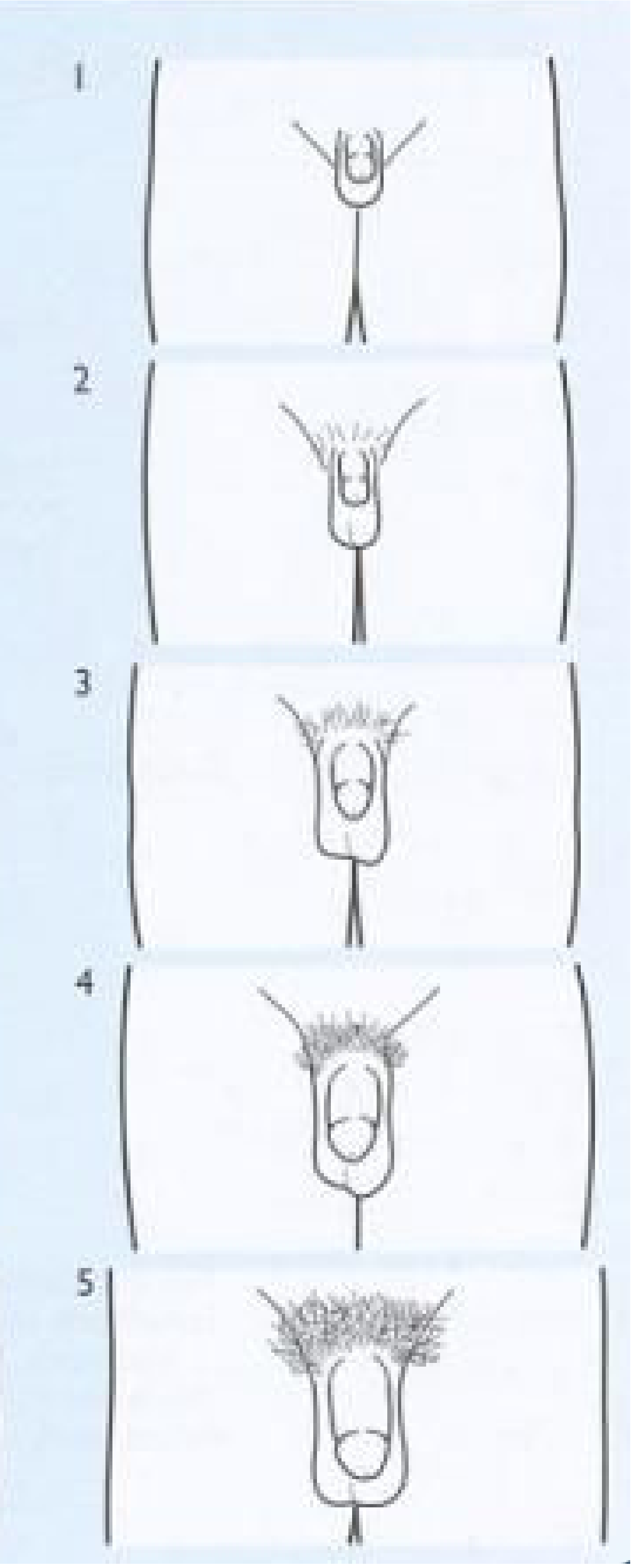

Which image represents your current physical features?

Please write one number. ______________________

How tall are you?<br>Please answer as best as you know your height	__________________________________
How much do you weigh?<br>Please answers best as you know your weight	__________________________________
When was your visit at the doctor's with the above mentioned pain?	◯ Today<br>◯ About a week ago<br>◯ 2-4 weeks ago<br>◯ More than a month ago
Now we have reached the last question:<br>When are you completing this questionnaire?<br><br>You may now press: submit.	◯ Before seeing the doctor<br>◯ After having seen the doctor<br>◯ I started completing the questionnaire before seeing the doctor, but competed it after having seen the doctor
